# *SPINK2* silencing suppresses leukemic proliferation and restores myeloid commitment via *MECOM* downregulation in acute myeloid leukaemia

**DOI:** 10.1038/s41420-026-02988-1

**Published:** 2026-03-03

**Authors:** Antonio Benedetto Ventura, Tiziana Loconte, Amer Ahmed, Lucia Deligio, Antonio Negri, Gabriella D’Angelo, Daria Di Molfetta, Pierre Cauchy, Barbara Mandriani, Xiao Zhang, Crescenza Pasciolla, Antonello Rana, Angela Iacobazzi, Giacomo Loseto, Mauro Cives, Luigi Viggiano, Francesco Massimo Lasorsa, Attilio Guarini, Maria Carmela Vegliante, Sabino Ciavarella, Giancarlo Castellano, Giuseppe Fiermonte, Giacomo Volpe

**Affiliations:** 1 Hematology and Cell Therapy Unit, IRCCS Istituto Tumori “Giovanni Paolo II”, Bari, Italy; 2 Department of Bioscience, Biotechnology and Environment, University of Bari “Aldo Moro”, Bari, Italy; 3 Department of Interdisciplinary Medicine, University of Bari “Aldo Moro”, Bari, Italy; 4 Max Planck Institute of Immunobiology and Epigenetics, Freiburg, Germany; 5 Experimental Pharmacology Unit, Istituto Nazionale Tumori Fondazione G. Pascale – IRCCS, Naples, Italy; 6 State Key Laboratory for Diagnosis and Treatment of Severe Zoonotic Infectious Diseases, Key Laboratory for Zoonosis Research of the Ministry of Education, Institute of Zoonosis and College of Veterinary Medicine, Jilin University, Changchun, China; 7 SJD Pediatric Cancer Center Barcelona, Institut de Recerca Sant Joan de Déu (IRSJD), Esplugues de Llobregat, Barcelona, Spain; 8 Hematology Section, Fondazione IRCCS Cà Granda, Ospedale Maggiore Policlinico, Milan, Italy; 9 Department of Pharmacy – Pharmaceutical Sciences, University of Bari “Aldo Moro”, Bari, Italy

**Keywords:** Acute myeloid leukaemia, Mechanisms of disease

## Abstract

Myeloid leukaemias harboring complex karyotypes present several unrelated cytogenetic abnormalities and form a distinct subset of AML linked to a dismal prognosis. Currently, no effective options are available for the treatment of those patients, and the discovery of novel therapeutic strategies represent an urgent clinical priority. We previously developed a bioinformatic framework for the identification of novel molecular vulnerabilities for disease stratification and treatment and observed SPINK2, a serine protease inhibitor Kazal-type 2, as a novel and promising candidate target in AML, with particularly pronounced effects in complex karyotype patients. Using publicly available bulk and single cell RNA-seq datasets, we discovered a robust association between *SPINK2* and cell cycle regulators, most notably S-phase genes. By performing shRNA-mediated genetic manipulation of *SPINK2* expression in a complex karyotype AML cell lines, we observed a profound impairment of proliferation coupled with an induction of terminal myeloid commitment. Moreover, *SPINK2*-deficient FUJIOKA cells revealed a significant association between *SPINK2* and *MECOM* expression, consistent with findings in patients harbouring complex karyotypes, yet absent in other AML subsets from the TARGET-AML cohort. Our findings suggest a novel potential correlation between *SPINK2* and *MECOM* expression in complex karyotype leukemias and warrant further investigation into the underlying molecular mechanisms through which the SPINK2-MECOM axis enforces aberrant self-renewal and the development of novel targeted approaches aimed at modulating its expression in complex karyotypic AML.

## Introduction

Acute myeloid leukaemia (AML) is an aggressive and molecularly heterogeneous malignancy of the hematopoietic system, marked by the accumulation of immature myeloid blasts in the bone marrow and peripheral blood [1–4]. While recent therapeutic advances have improved outcomes in given patient subgroups, standard induction regimens have changed little over the past three decades [5]. This therapeutic plateau is largely attributable to the diverse and evolving genomic landscape of AML, which drives distinct biological behaviours and differential treatment responses [6–8]. The integration of high-throughput sequencing and advanced computational tools has facilitated the discovery of recurrent mutations, such as FLT3-ITD, NPM1, and CEBPA, and their incorporation into risk stratification systems like the European LeukemiaNet (ELN) 2022 guidelines [9, 10]. Yet, a substantial unmet need remains for biomarkers that can further refine prognostic models and inform targeted therapeutic strategies. Beyond mutational profiling, transcriptomic and proteomic approaches have expanded the biomarker discovery landscape, enabling the identification of molecular signatures associated with disease subtypes, therapeutic response, and survival. Gene expression-based classifiers have shown particular promise in stratifying AML subgroups [6, 11, 12], while machine learning frameworks have demonstrated the capacity to integrate diverse omics datasets to uncover novel predictive markers [13, 14]. These integrative approaches are essential for navigating the complexity of AML and translating molecular insights into clinical benefit. Among emerging candidates, SPINK2, a serine protease inhibitor of the Kazal type 2 family, has recently emerged as a novel player in haematopoietic fate transition from the hemogenic endothelium [15] and has also been proposed as an independent predictor of dismal prognosis in AML [16–18]. Elevated SPINK2 expression correlates with resistance to induction therapy and increased relapse risk, particularly within NPM1-mutant and cytogenetically intermediate-risk groups. Transcriptomic analyses suggest that SPINK2 may influence leukemogenesis through regulation of ferroptosis and immune response pathways [17]. Notably, in paediatric AML, SPINK2 has been implicated as a marker of primary induction failure in patients harbouring NUP98 rearrangements [19]. We recently developed a computational pipeline designed to identify novel biomarkers by analysing publicly available transcriptomic datasets. Through this approach, we uncovered several candidate genes with potential clinical relevance, such as *GFI1* for which we demonstrated a strong association with a FLT3-ITD molecular signature in cytogenetically normal AML [20], and *WBP5*, whose elevated expression correlated with activation of the *HOX* gene cluster [12]. Importantly, in these settings we also observed *SPINK2* among the markers most significantly associated with inferior outcome. To assess this further, we conducted single-cell transcriptomic analysis of both healthy and leukemic bone marrow datasets observing *SPINK2* being specifically enriched in CD34⁺ quiescent hematopoietic progenitors under physiological conditions, while remaining highly expressed in proliferative AML cells. Functional silencing of *SPINK2* in an AML cell line characterized by its high expression led to markedly impaired leukemic cell growth being also accompanied by a boost in myeloid maturation, highlighting its potential as a therapeutic vulnerability. Transcriptomic analysis following *SPINK2* ablation revealed a downregulation of genes involved in cell cycle progression and a strong association with MECOM, a known transcription factor involved in leukemia. This relationship was further confirmed using publicly available data from the TARGET-AML cohort. Collectively, these findings highlight the *SPINK2*-*MECOM* axis as a potentially relevant molecular route for therapeutic targeting in AML.

## Results

### *SPINK2* is expressed in immature non-proliferative haematopoietic cells

Our previous work based on the generation of a bioinformatic framework for the unbiased identification of novel indicators of prognosis and survival outcome in patients with AML [12], highlighted SPINK2 as a potential prognosticator and targetable vulnerability for the treatment of such disease. Our analysis, initially based on the use of microarray data from the Verhaak cohort [21], indicated that higher *SPINK2* levels were largely correlated with poor cytogenetic risk, were highest in patients carrying complex karyotypes and associated with inferior survival (Figure S1A-E), in keeping with previous work associating upregulated *SPINK2* levels to AML. Such a trend was also determined when performing the same stratification into *SPINK2* high versus low expressers using both TCGA [22] and TARGET-AML [23] cohorts, determining a strong and statistically significant association of *SPINK2* levels with inferior outcomes (Figure S1F-G). Analysis of publicly available cancer data through TNMplot [24] revealed that *SPINK2* levels were largely upregulated in AML versus healthy bone marrow, whereas no differences were seen when comparing *SPINK2* levels between other cancers and the respective healthy tissue, with the exception of testicular cancer in which an opposite trend was observed (Figure S2A).

**Fig. 1:**
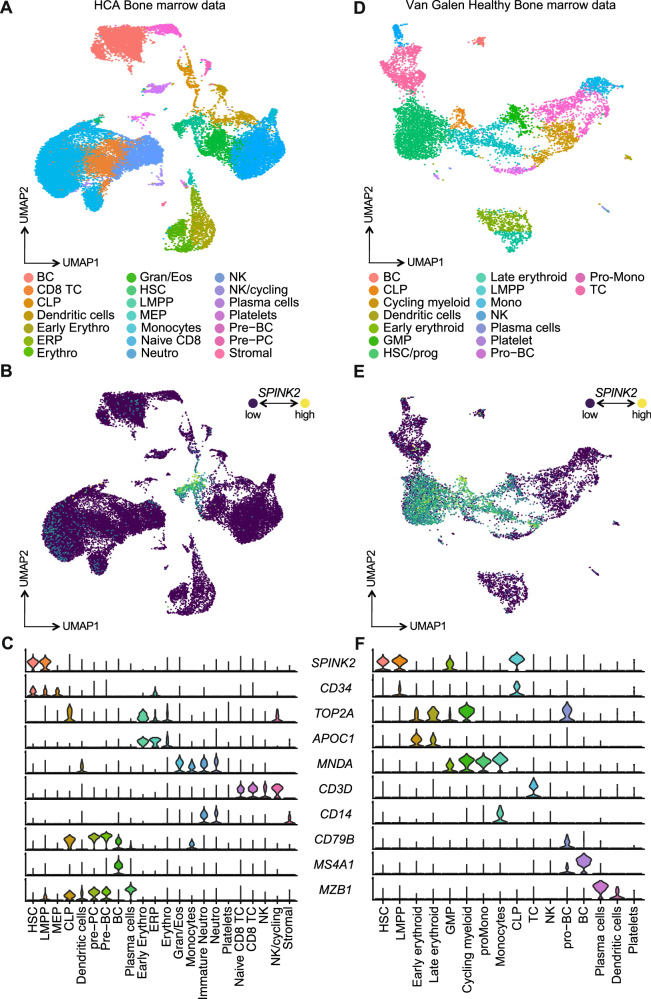
*SPINK2* expression is restricted to CD34^+^ cells in human healthy bone marrow. **A** Uniform manifold approximation and projection (UMAP) representation of single cell RNA-seq data of healthy bone marrows from the Human Cell Atlas (HCA, left) and (**D**) from the Van Galen dataset. Clusters are coloured by the cell types indicated in the legend at the bottom of the plot. **B** UMAP representation of *SPINK2* expression in HCA and (**E**) Van Galen datasets. **C** Violin plots showing the expression of *SPINK2* and the canonical markers used to reannotate the clusters shown in panel A in the HCA and (**F**) the Van Galen dataset.

We set out to investigate the potential role on *SPINK2* in both healthy and aberrant haematopoiesis. In this endeavour, we first explored the single cell RNA-sequencing dataset of the Human Cell Atlas [25] to determine the expression behaviour of *SPINK2* in freshly isolated bone marrow cells from healthy individuals. By reclustering and manually reannotating all cell populations found within the dataset, we observed *SPINK2* expression to be mostly confined to the immature fraction of the bone marrow (Fig. 1A and B), that is haematopoietic stem cells (HSCs) and lymphoid-primed multipotent progenitors (LMPPs), also displaying a substantial overlap with the expression of *CD34*. Conversely, little or no expression was found along either myeloid or lymphoid maturation trajectories (Fig. 1C). To corroborate those observations, we also retrieved and inspected the dataset reported by Van Galen et al., which encompasses both healthy and leukaemic bone marrow specimens [26]. Analysis of healthy haematopoietic cells revealed the highest levels of *SPINK2* to be found in the most immature clusters, those being HSCs, LMPPs as well as granulocytic-monocytic progenitors (GMPs) and common lymphoid progenitors (CLPs). Similarly, the expression of *SPINK2* was markedly downregulated in more committed cells, being completely absent in mature cells (Fig. 1D-F). Importantly, we observed no evident correlation between *SPINK2* levels, and the expression of cell cycle related genes, suggesting that *SPINK*2 is associated with a quiescent or low-proliferative status (Fig. 1C and F and Figure S1).

### *SPINK2* is associated with a proliferative leukemic phenotype

**Fig. 2:**
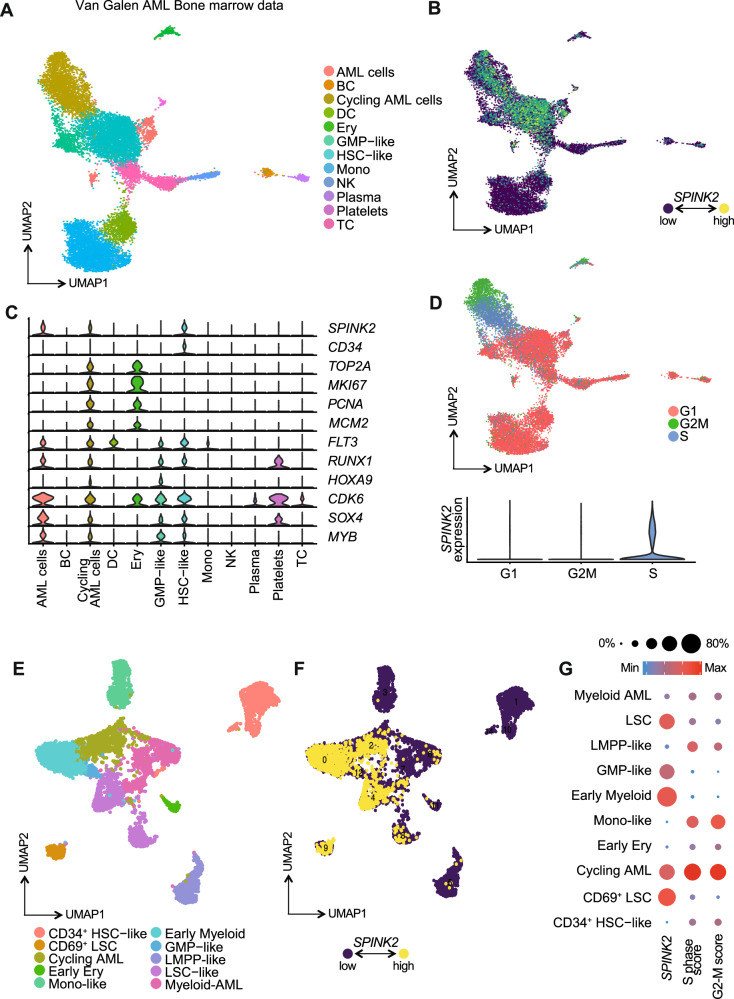
*SPINK2* expression associates with cycling AML cells in leukemic bone marrow. **A** UMAP representation of single cell RNA-seq data from the Van Galen leukemic cohort. Clusters are coloured by the cell types indicated in the legends on the righthand side of the plot. **B** UMAP representation of *SPINK2* expression in the leukemic samples from the Van Galen cohort. **C** Violin plots showing the expression of *SPINK2* and other canonical markers typically observed in leukemia. **D** UMAP representation of cell cycle module expression in the Van Galen leukemic dataset. Cells are coloured according to cell cycle phase as indicated in the legend on righthand side. The violin plot at the bottom indicates the level of *SPINK2* expression in each cell cycle phase. **E** UMAP representation of reclustered AML blast and blast-like only from the Van Galen dataset. Clusters are coloured by the cell types indicated in the legends displayed at the bottom of the plot. **F** UMAP representation of *SPINK2* expression in the reclustered blast and blast-like cells from the Van Galen cohort. **G** Bubble plot showing the expression of *SPINK2* as well as the S-phase score and the G2-M score for each cluster indicated in the UMAP in **E**.

We next sought to assess the functional role of *SPINK2* in AML by reanalysing publicly available single cell transcriptomic profiles from a cohort of 16 leukaemic bone marrow specimens reported by Van Galen and coworkers [26] to determine the expression of *SPINK2* in leukemic cells (Fig. 2A). In agreement with the observation obtained with healthy specimens, we detected *SPINK2* in the HSC-like cluster, also being accompanied by substantial *CD34* co-expression. In keeping with the HCA dataset, *SPINK2* appeared largely undetectable or expressed at a very low level in more committed cells (Fig. 2B, C). Importantly, we also observed its expression in the clusters annotated as AML and cycling AML cells, together with genes typically expressed in leukaemic cells, such as *FLT3*, *CDK6*, *SOX4, RUNX1, HOXA9* and *MYB*. Unlike healthy cells, we observed that *SPINK2* was co-expressed with cell cycle associated genes, including *TOP2A* and *MKI67*, and was particularly correlated with S-phase related genes, such as *PCNA*, *MCM2* and others (Fig. 2C, D). To assess whether *SPINK2* expression was uniformly distributed across AML-like cellular subsets, we reclustered only AML blast and blast-like populations after excluding all residual normal hematopoietic and differentiated cells (Fig. 2E-F). While *SPINK2* expression was detectable across most malignant subsets, its levels were markedly heterogeneous among the most immature compartments: it was strongly enriched in LSC-containing clusters and early myeloid progenitors but nearly absent in CD34⁺ HSC-like cells and in more differentiated myeloid AML populations (Fig. 2G). Notably, *SPINK2* expression was also elevated in cycling AML cells. Together, these findings show that *SPINK2* enrichment is not uniform across AML subtypes and is instead closely associated with cycling AML populations. These findings suggest that *SPINK2* expression may be linked to cell cycle progression in leukaemic cells.

### *SPINK2* knock-down attenuates leukaemic proliferation and boosts myeloid maturation in vitro

**Fig. 3:**
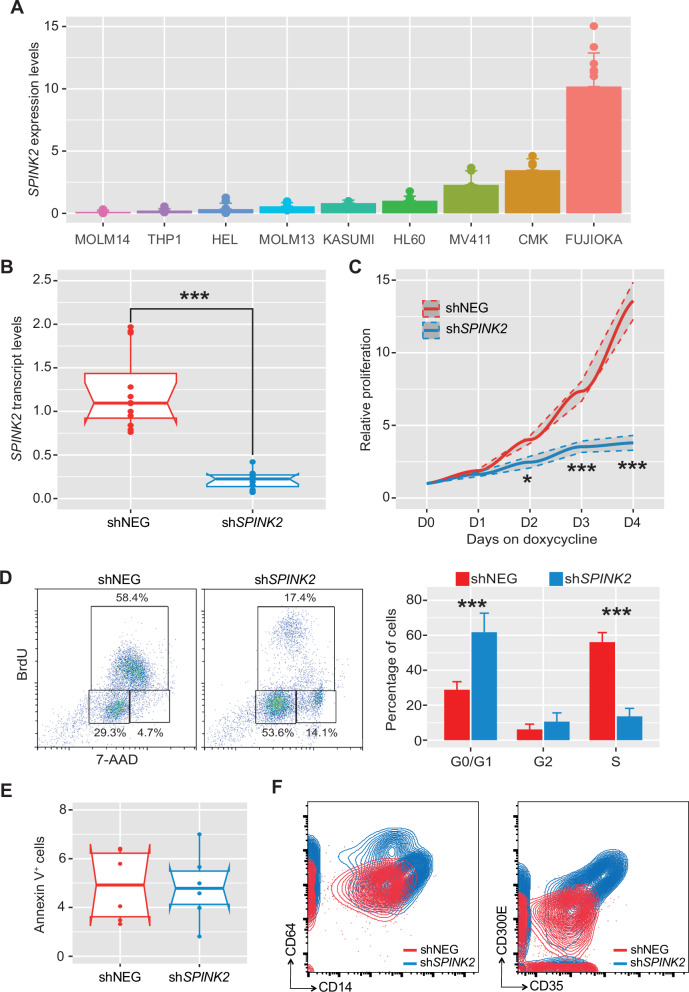
*SPINK2* ablation reduces leukemic proliferation and boosts commitment to myeloid maturation. **A** Barplot showing the expression of *SPINK2* measured by qPCR in a panel of AML cell lines. *n* = 6 for each cell line. **B** Bowtie plot representing *SPINK2* mRNA quantification by q-PCR following *SPINK2* shRNA silencing and normalized using GAPDH (*n* = 6). **C** Lineplot displaying cell viability upon *SPINK2* knock-down determined by counting cells every 24 h for four consecutive days post shRNA induction. The results are indicative of 3 independent experiments (*n* = 6). **D** Two-dimensional flow cytometric dot plot showing BrdU incorporation 72 hours post doxycycline induction. The three gates indicate the G0/G1, S and G2/M phases of the cell cycle. The barplot on the righthand side represent an average of three independent experiments (*n* = 6). **E** Bowtie plot showing the percentages of Annexin V^+^ apoptotic/necrotic cells. **F** Representative two-dimensional flow cytometric contour plots showing the expression levels of CD14, CD64, CD35 and CD300E in FUJIOKA cells that have undergone *SPINK2* silencing 96 hours post doxycycline induction (*n* = 6). The statistical analysis reported in this figure have been performed using student’s t test (***p < 0.001, *p < 0.05).

Our observation of *SPINK2* expression being correlated with cell-cycle related genes prompted us to investigate whether manipulating its expression would result in a proliferation arrest. For this purpose, we measured by quantitative RT-PCR the expression of *SPINK2* in different AML cell lines available in our laboratory, and determined that *SPINK2* is least expressed in MOLM14 and THP1 cells and most highly expressed in FUJIOKA cells (Fig. 3A). This latter cell line is known to hold TP53 mutations and appear to display a mutational burden more closely resembling that of complex karyotype leukemias [7]. Therefore, we established a doxycycline-inducible cell line by transducing FUJIOKA cells with lentiviral particles encoding either a scrambled control or a *SPINK2* shRNA, all under the regulation of a Tet-responsive element. Stably transduced cells were cultured under continuous administration of doxycycline for shRNA activation and, to verify the efficient knock-down of *SPINK2*, cells were collected 48 hours after doxycycline induction for qPCR analysis, demonstrating an almost complete ablation of *SPINK2* mRNA expression (over 90%) (Fig. 3B). We cultured proficient and deficient cells for four consecutive days counting cells daily to assess how *SPINK2* silencing would impact on their proliferative capacity. In these experimental settings *SPINK2* knock-down was accompanied by a sustained and statistically significant proliferative retardation, this becoming particularly pronounced at 72 hours post silencing induction (Fig. 3C). We then conducted a flow cytometric BRDU incorporation analysis at the time points in which the proliferative defect became more evident, that is, at 72 hours post doxycycline induction. At this stage, deficient cells displayed a substantial accumulation in the G0/G1 phase of the cell cycle with a concomitant reduction of cells progressing through the S phase and G2/M phases, thus suggesting that *SPINK2* depletion impairs proper cell cycle progression, primarily resulting in G0/G1 arrest (Fig. 3D). We also assessed whether this growth retardation defect could be ascribed to an increased rate of apoptosis being stimulated by *SPINK2* ablation. In this respect, we measured the percentage of cells displaying Annexin V^+^ staining at 72 hours post doxycycline induction, although no noticeable difference was detected (Fig. 3E), indicating that such proliferation defect is not dependent on cells undergoing apoptosis but would rather be due to a cell cycle arrest.

Ultimately, we tested whether *SPINK2* suppression could alleviate the myeloid maturation block typically observed in leukemia. To this end, we collected the doxycycline-induced cells at 96 h and stained them with antibodies against canonical myeloid surface markers CD14, CD35, CD64 and CD300E by flow cytometry (Fig. 3F). Remarkably, *SPINK2* knock-down led to a robust and consistent upregulation of all tested markers, making deficient cells progressing towards a more differentiated myeloid state. This shift in the immunophenotype suggests that *SPINK2* could also act as a molecular barrier to myeloid maturation in leukemic cells, thus supporting the maintenance of a more immature and highly proliferative state.

### *SPINK2* ablation rewires the transcriptome towards a more quiescent and differentiation-skewed state

**Fig. 4:**
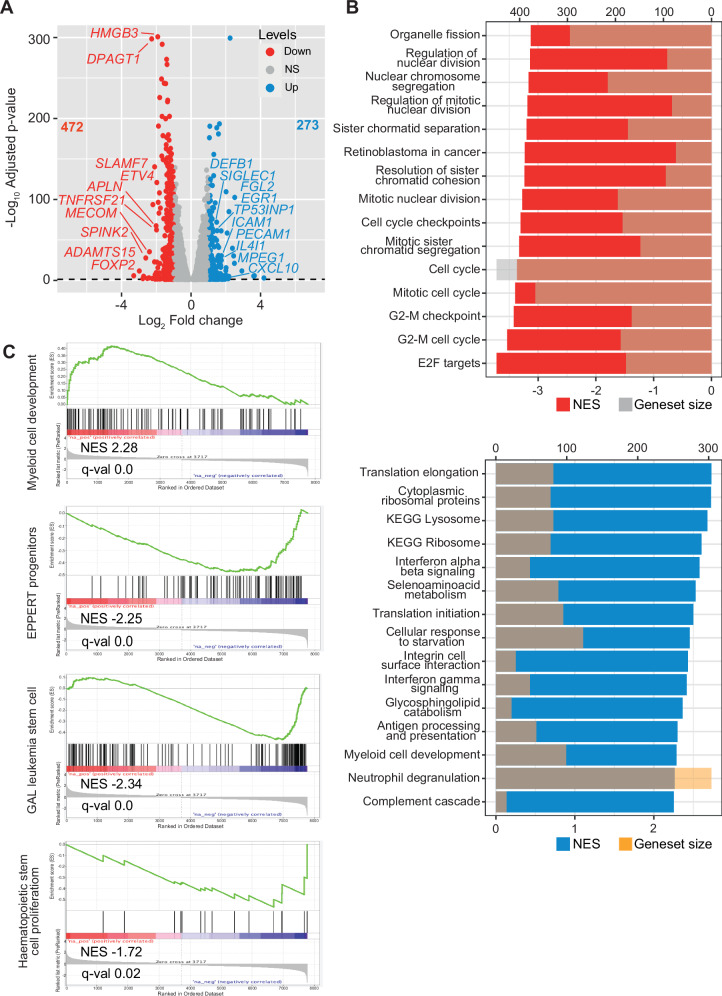
Transcriptomic profiling of *SPINK2* ablation reveals differential regulation of cell cycle and myeloid maturation programs. **A** Volcano plot showing differentially regulated genes upon *SPINK2* mRNA ablation. Downregulated (*n* = 472) and upregulated (*n* = 273) genes are indicated with red and blue dots, respectively, while non-significant genes are indicated in grey. **B** Shaded barplot indicating either the negative (top, in red) or positive (bottom, in blue) normalized enrichment score for the indicated GSEA terms for the downregulated or upregulated genes indicated in panel A, while shaded bars indicate the size of the geneset. **C** Representative GSEA signatures for upregulated (first panel) or downregulated genes following *SPINK2* silencing.

In order to obtain a global view of the molecular consequences associated to *SPINK2* depletion in FUJIOKA cells, we performed bulk RNA-sequencing comparing proficient versus deficient cells using a Log_2_ fold change cut-off of +/- 1 and considering as significant those with a q-value below 0.05. Using those criteria, differential gene expression analysis revealed widespread changes in transcriptomic profiles, with a total of 472 downregulated and 273 upregulated genes upon *SPINK2* knockdown (Fig. 4A).

Among the most downregulated genes, we identified *MECOM*, *ETV4*, *APLN*, *HMGB3* and *FOXP2*, among others, all of which have been implicated in oncogenic process and disease progression [27–31]. In contrast, among the most upregulated genes we observed *PECAM1*, *SIGLEC1*, *CXCL10*, *EGR1* and *FGL2*, whose expression has been consistently associated with a more mature myeloid state and differentiation programs [32–36].

To gain a comprehensive understanding of the biological processes and pathways affected by *SPINK2* silencing, we performed gene set enrichment analysis (GSEA) using a wide range of curated gene sets, including collections from Gene Ontology (GO) Biological Processes, WikiPathways, BioCarta, Reactome, and the Hallmark gene sets from MSigDB, among others. Among the downregulated genes, we observed an overwhelming enrichment for gene sets associated with cell cycle regulation and mitotic processes. In particular, terms related to mitotic nuclear division, sister chromatid segregation, spindle assembly checkpoint regulation, and G2/M phase progression were significantly depleted. Additionally, several E2F transcription factor targets, that are crucial regulators of G1/S transition, were notably repressed, suggesting impaired activation of proliferative programs. Consistent with these observations, pathways involved in G2/M checkpoint signalling, DNA replication initiation, and mitotic spindle organization also showed significant downregulation across multiple databases (Fig. 4B). Importantly we also observed a strongly significant positive association with genes that are part of the term “myeloid cell development”, in keeping with the observation of FUJIOKA cells undergoing a myeloid boost in response to *SPINK2* knock-down (Fig. 4C).

Conversely, gene sets related to protein synthesis and immune responses were significantly enriched among the upregulated genes. Prominent terms included cytoplasmic ribosomal protein translation, initiation and elongation of translation, interferon signalling, and antigen processing and presentation. These findings were consistently observed across multiple pathway collections, further supporting the activation of stress and immune-related responses (Fig. 4B). Collectively, these data demonstrate that *SPINK2* silencing disrupts core cell cycle-related gene networks while triggering compensatory upregulation of translational and immune pathways, highlighting its key role in sustaining proliferative transcriptional programs in FUJIOKA cells while suppressing those related to terminal myeloid maturation.

### *SPINK2* ablation associates with *MECOM* downregulation

**Fig. 5:**
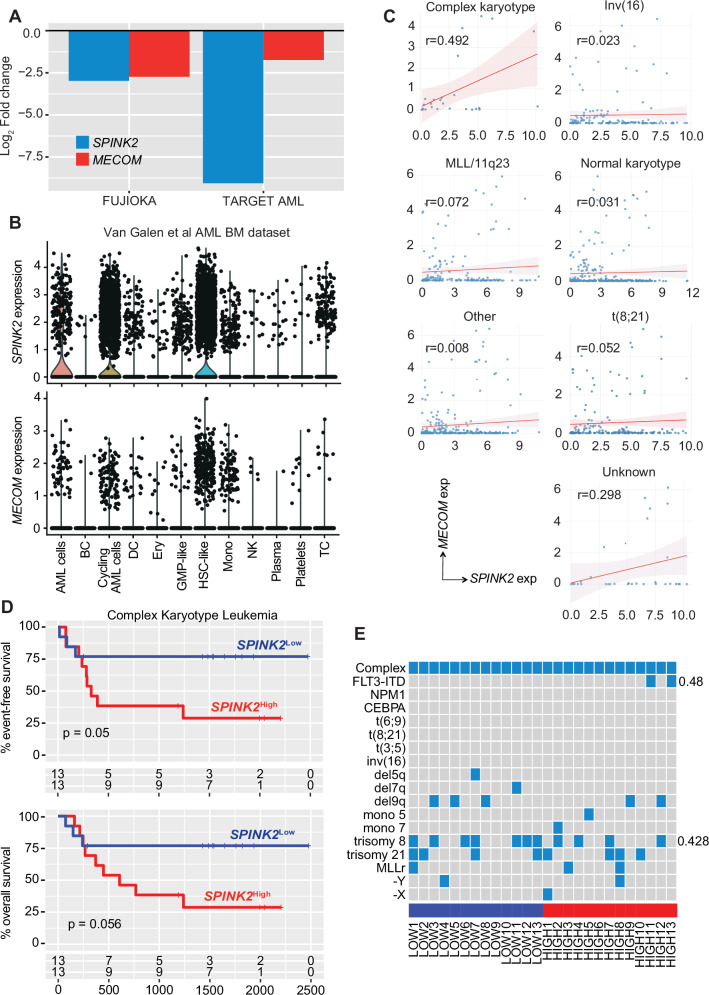
*SPINK2* ablation correlates with *MECOM* in complex karyotype leukemia. **A** Barplot showing the expression level of *SPINK2* and *MECOM* in FUJIOKA cells and in the TARGET-AML cohort. **B** Violin plot reporting the expression levels of *SPINK2* (top) and *MECOM* (bottom) in all annotated cell population from the scRNA-seq dataset of the AML Van Galen cohort. **C** Line plot showing the correlation between *SPINK2* and *MECOM* in different subclasses of TARGET-AML cohort. The Pearson R value is indicated in each plot. **D** Kaplan-Meyer curves reporting the overall (top) and event-free survival (bottom) of *SPINK2* low and high expressers from the complex karyotype subcohort. **E** Oncoprint heatmap showing the association of specific mutations to *SPINK2* expression levels in complex karyotype high and low *SPINK2* expressers.

The inspection of the transcriptomic reshaping in response to *SPINK2* silencing also revealed *MECOM* as one of the most significantly downregulated and correlated genes in FUJIOKA (Log_2_ FC -2.73, q < 0.0001) (Fig. 5A). Notably, analysis of scRNA-seq data from the Van Galen cohort, despite the low overall expression and the limited and heterogeneous sample size, also showed that *MECOM* expression was enriched in cells exhibiting the highest levels of *SPINK2*, those being cycling AML cell and HSC-like cells (Fig. 5B). To corroborate these observations, we looked for the expression levels of *SPINK2* and *MECOM* and their correlation in the TARGET-AML cohort, as this is the largest and most extensively annotated dataset with all clinical features. We noted that, when performing the dichotomization of all patients on the basis of *SPINK2* expression (*SPINK2*^high^ top quartile, *SPINK2*^low^ bottom quartile), we observed a significant association of *SPINK2* (Log_2_ FC -9.05, q < 0.0001) and *MECOM* (Log_2_ FC -1.74, q < 0.0001) (Fig. 5A). Importantly, correlation analysis showed a moderate yet highly significant positive association between those two genes, this being confined in AML patients harbouring complex karyotype lesions only (R^2^ = 0.49, p = 0.017), while no correlation was evident for any of the other cytogenetic subgroups reported in this cohort (Fig. 5C). Building on this observation, we then focused exclusively of those AML cases that were characterized by complex karyotype lesions and performed a dichotomization based on *SPINK2* levels; due to the limited sample size, we applied a median cutoff (13 Low vs. 13 High), and although both overall and event-free survival analysis did not reach statistical significance (p = 0.056 and 0.05), the same adverse trend for the High group was retained (Fig. 5D), with no statistically significant associations emerging between *SPINK2* levels and specific mutational patterns (Fig. 5E).

Our results indicate a potential correlation whereby *SPINK2* may influence or co-regulate *MECOM* dependent transcriptional programs that underpin aberrant cell cycle dynamics in high-risk AML.

## Discussion

In the present study, we have investigated the role of *SPINK2* in the context of AML, and we have identified it as a previously underappreciated regulator of both cell cycle progression and myeloid maturation blockade in leukemic cells. Our findings extend current knowledge on *SPINK2* by demonstrating its dual contribution to sustaining proliferative capacity while maintaining cells in an immature state, thereby positioning it as a potentially central driver of leukemogenesis. We have previously designed a computational pipeline for the identification of novel biomarkers of disease stratification in AML through which SPINK2 emerged as one of the most significant predictor of inferior survival. In keeping with previous reports describing elevated *SPINK2* levels in bone marrow samples of patients characterized by various forms of AML and of its correlation with dismal prognosis [16, 18, 37, 38], we confirmed its prognostic significance in other cohorts using publicly available datasets from the TCGA-LAML [22] and TARGET-AML [23] studies. This observation further underscores its potential as a prognostic marker of adverse outcome. Notably, this prognostic value appears to be independent of other established risk stratification factors, as such highlighting the value of SPINK2 as a novel candidate for therapeutic targeting in aggressive leukemias.

In our study we have made use of publicly available single-cell RNA-seq data of healthy human bone marrow specimens from either the Human Cell Atlas [25] or from the Val Galen study [26] and we observed that *SPINK2* expression is tightly restricted to the most immature CD34⁺ hematopoietic stem and progenitors populations, suggesting a key role in the regulation of early hematopoietic programs. Such a restricted expression pattern aligns with previous work demonstrating that *SPINK2* is among the key genes, together with *RUNX1*, *HOXA9*, *MLLT3*, *MECOM* and *HLF*, that allows a reliable discrimination of true nascent HSCs during human gestation, being a distinctive early markers of such cells emerging from the hemogenic endothelium [15]. Thus, SPINK2 belongs to a small set of “stemness-defining” genes that are essential for the earliest stages of hematopoietic ontogeny. Importantly, whereas most progenitor-associated transcripts are normally switched off as differentiation proceeds, AML cells exhibited not only the persistence but also an enrichment of *SPINK2* expression within actively cycling populations. Moreover, its transcript levels showed a strong positive correlation with the expression of several genes associated with S phase progression, including critical DNA replication and repair programs essential for cell proliferation and genomic stability. This observation suggests that leukemic cells may hijack.

In order to experimentally prove the functional relevance of *SPINK2*, we screened a panel of AML cell lines available in our laboratory and identified FUJIOKA cells as the most suitable model due to their highest *SPINK2* endogenous expression. We then generated a doxycycline-inducible system to acutely suppress *SPINK2* in a controllable manner, with which we demonstrated that ablation of *SPINK2* expression is accompanied by a marked reduction in proliferation. This phenomenon is not attributable to cell death induction, given that no difference in terms of apoptosis was noticeable, but rather due to cells undergoing a G0/G1 phase arrest, as shown by BRDU incorporation studies, indicating that *SPINK2* is indispensable for cell cycle progression beyond this point. Remarkably, *SPINK2* knockdown resulted in the upregulation of several myeloid differentiation markers such as CD14, CD64, CD35, and CD300E, suggesting that *SPINK2* is not only involved in promoting leukemic cell proliferation but also in sustaining an immature, undifferentiated state by impeding myeloid maturation. Together, these data suggest that SPINK2 acts as a molecular switch that couples a proliferative drive with a myeloid commitment blockade, a hallmark feature of AML biology [39, 40].

To gain some mechanistic insights, we performed bulk transcriptomic profiling of FUJIOKA cells upon *SPINK2* ablation by bulk RNA-sequencing. This revealed a global downregulation of genes that are critical for cell cycle control, in particular those orchestrating mitotic entry, DNA replication, and chromatid segregation. Among the most significantly downregulated genes, we identified *MECOM* (EVI1), a well-characterized transcription factor implicated in AML pathogenesis [41, 42]. Consistent with the observation that *MECOM*, alongside *SPINK2*, is one of the key genes defining nascent human HSCs [15], *MECOM* is also known to maintain stem cell self-renewal [27, 43, 44], inhibit myeloid differentiation [45], and promote leukemic cell proliferation, those being functions that closely mirror the phenotype observed upon *SPINK2* knockdown in our model system. Moreover, MECOM has been shown to directly regulate genes involved in cell cycle control [43], including G1/S checkpoint regulators [46], suggesting a potentially convergent mechanism with SPINK2.

The parallel regulation of leukemic stemness and cell cycle programs by both SPINK2 and MECOM raises the possibility of a functional interaction between those two genes. MECOM has been shown to directly regulate G1/S checkpoint genes, suggesting that its downregulation upon *SPINK2* silencing may represent a convergent mechanism by which proliferative arrest is imposed. Although we cannot establish whether SPINK2 could act upstream, downstream or in parallel with MECOM, the correlation suggests that these two factors may participate in a shared regulatory axis essential for sustaining the leukemic state, for which more dedicated studies are required in order to map the molecular circuitries connecting these two genes. Furthermore, it will be important to explore whether SPINK2 influences MECOM at the transcriptional, post-transcriptional or epigenetic level.

Beyond mechanistic implications, our findings could have therapeutic relevance. The identification of SPINK2 as a determinant of leukemic cell cycle progression and differentiation blockade opens new avenues for targeted intervention, in particular for certain subclasses for which limited options are available, such as complex karyotype leukemias. Current AML therapies primarily exploit vulnerabilities in proliferation or metabolic dependencies, although therapeutic resistance and relapse are quite common, often due to the persistence of leukemic stem cells that evade therapy through quiescence of alternative survival pathways. By targeting SPINK2, it could be possible to simultaneously impair proliferative drive and resolve the myeloid commitment barrier, thereby eradicating both cycling blasts and quiescent progenitor-like AML cells. It is worth considering that, the restricted expression of *SPINK2* in healthy CD34^+^ hematopoietic cells suggests that inhibition of *SPINK2* could be well tolerated in the adult hematopoietic system. Nevertheless, given the essential role of SPINK2 in nascent HSCs during development, future work will need to carefully assess the potential consequences of *SPINK2* targeting on normal hematopoiesis in preclinical models.

In summary, our work uncovers SPINK2 as a novel contributor to leukemogenesis, functioning at the intersection of cell cycle progression, stemness maintenance and differentiation blockade. By uncovering its association with poor prognosis, its enrichment in cycling AML populations, and its functional requirement for proliferation and differentiation arrest, we establish SPINK2 as both a biomarker of adverse outcome and a candidate therapeutic target. Moving forward, efforts should focus on dissecting the mechanistic interplay between SPINK2 and established oncogenic regulators such as MECOM, exploring the feasibility of pharmacologic inhibition, and validating its role in in vivo leukemia models. Collectively, these findings expand our understanding of leukemic biology and provide a rationale for the development of SPINK2-directed strategies to improve outcomes in high-risk AML subsets.

## Materials and Methods

### Cell lines

The FUJIOKA cell line was obtained from the Japanese Collection of Research Bioresources Cell Bank. Cells were cultured in RPMI-1640 medium supplemented with 10% foetal bovine serum (FBS), 50U/ml penicillin, 50 µg/ml streptomycin, 2mM L-glutamine. Cells were kept at a concentration of 0.5 × 10^6^ cells/ml at 37 °C under normoxic conditions with 5% CO_2_ in a humidified incubator and were washed with PBS between passages. The FUJIOKA cell line has been routinely tested for mycoplasma.

### Availability of transcriptomic datasets

Available single cell RNA-sequencing integrated and pre-processed data from 8 bone marrow donors reported in Hay et al. were retrieved in the Human Cell Atlas databased (https://preview.data.humancellatlas.org) [25]. Data from both healthy and leukemic bone marrow reported in Van Galen et al. were downloaded from Gene expression Omnibus under the GSE116256 accession number [26]. Normalized data were projected using Uniform Manifold Approximation and Projection (UMAP) to visualize cell types clustered by transcriptional similarities. Cell clusters were manually annotated on the basis of the expression of canonical markers. Bulk RNA-seq data from the TCGA, Beat-AML, and TARGET-AML cohorts were downloaded from Genomic Data Commons under the dbGaP study accession numbers phs000178 (TCGA-LAML) [22], phs1657 (Beat-AML) [47], and phs000465.v18.p7 (TARGET-AML) [23], respectively.

### Generation of a stable shRNA-expressing FUJIOKA cell line, proliferation, apoptosis and differentiation assay

For the shRNA silencing work, stable shRNA expressing cell lines were generated. For this purpose, we interrogated the GPP Web Portal (https://portals.broadinstitute.org/gpp/public/gene/search_clones) and obtained the shRNA that displayed the highest predicted silencing score: shRNA1, **TCAGGGAAGGTGGTCATAATA** (TRCN0000373635). The shRNA was cloned into the Tet-pLKO-puro vector (Addgene plasmid #21915) using AgeI/EcoRI restriction sites [48]. The integrity of the shRNA insert was confirmed by sequencing. A scrambled control shRNA plasmid (shCtr), Tet-pLKO-puro-Scrambled (Addgene plasmid #47541), was used as a non-targeting control [49]. Recombinant lentiviral particles were generated by transient transfection of 293 T cells as previously described [50], used for standard spinoculation protocols using polybrene (TR-1003-G, Merck) at the end of which transduced cells were selected with puromycin (A1113803, Gibco).

Puromycin-resistant cells were seeded at a density of 10^6^ cells/ml and administered continuously with doxycycline (1 µg/µl) cells for shRNA induction for the duration of the experiment. Viable cells were counted and passaged at a 1:2 ratio of day for four consecutive days to determine their growth rate. Cell cycle analysis was carried out by labelling cycling cells with 10 µM BRDU (8811-6600-42, Thermofisher) for 1 hour, after which cells were co-stained with 7-AAD (IM3630c, Beckman-Coulter) according to manufacturer’s instructions, as previously described [3]. Apoptosis analysis was performed using an Annexin V kit following manufacturer’s instruction(130-092-052, Miltenyi Biotech). Myeloid maturation studies were performed using antibodies against CD14, CD35, CD64 and CD300E (Beckman Coulter). Acquisition of immunostaining data was carried out on a Cytoflex Instrument (Beckman Coulter) and the analysis was performed using a Flowjo software (Flowjo, LLC). *SPINK2* silencing efficiency was measured by qPCR on a CFX Duet Biorad Real Time PCR using the following oligos:

forw: 5’-AATCATTCGAAATGGACCC-3’

rev: 5’-TATCTAGTCTGCCAGTGAAG-3’.

### RNA-sequencing and bioinformatic analysis

RNA-seq library preparation was performed using Optimal Dual-mode mRNA Library Prep Kit (BGI-Shenzhen, China) according to manufacturer’s instruction. The generated DNA nanoballs were loaded in patterned nanoarray and PE150 base reads were generated by sequencing on a MGI G400 sequencing platform at MGI facility in Warsaw, Poland. Upon data generation, quality control of raw sequencing reads was performed using FastQC. Reads were aligned to the human reference genome (GRCh38) using STAR aligner. Gene-level counts were quantified using featureCounts and normalized using the DESeq2 package in an R environment. The differential gene expression analysis was performed using DESeq2 comparing *SPINK2*-deficient and proficient FUJIOKA cells, by applying a significance threshold of adjusted p-value (q-value) < 0.05 while the absolute Log2 fold change was set at > 1 for upregulated genes and < 1 for downregulated genes.

Gene Set Enrichment analysis (GSEA) was performed using the GSEA software (Broad Institute, Cambridge, MA) with 1000 gene set permutations. Multiple gene set collections were analysed including Gene Ontology (GO) Biological Processes, Wikipathways, BioCarta, Reactome and Hallmark gene sets from MSigDB. Gene sets with a false discovery rate (FDR) q-value < 0.05 and normalized enrichment score (NES) absolute value > 1.5 were considered significantly enriched, as previously reported [7]. The RNA-seq data generated in this study have been deposited on Gene Expression Omnibus with accession number GSE313901.

### Statistical analysis

All the in vitro experiments presented in this study were analysed using an unpaired two-tailed Student’s t-test, unless otherwise specified. Data are presented as mean ± standard deviation (SD), and the number of biological replicates (n) is indicated in the figure legends. The overall survival was evaluated by generating Kaplan-Meyer curves and the statistical significance was assessed by applying the log-rank (Mantel-Cox) test. For the Gene Set Enrichment Analysis both the normalized enrichment score (NES) and false discovery rate q-value were calculated using the GSEA software (Broad Institute, Cambridge, MA). A p value < 0.05 was considered statistically significant.

## Supplementary information

Supplementary Information
