# Supplementary material for: SPINK2 silencing suppresses leukemic proliferation and restores myeloid commitment via MECOM downregulation in acute myeloid leukaemia
Source: Cell Death Discov. 2026 Mar 3;12:135. doi: 10.1038/s41420-026-02988-1 (PMC13039488; doi:10.1038/s41420-026-02988-1)
Supplement: Supplementary file 1 — Supplementary Information [file 41420_2026_2988_MOESM1_ESM.pdf]

# *SPINK2* silencing suppresses leukemic proliferation and restores myeloid commitment via *MECOM* downregulation in acute myeloid leukaemia

Ventura AB [1,10], Loconte T[1,10], Ahmed A[2,10], Deligio L[1], Negri A[1], D'Angelo G[3], Di Molfetta D[2], Cauchy P[4], Mandriani B[5], Zhang Xiao[6], Pasciolla C[1], Rana A[1], Iacobazzi A[1], Loseto G[1], Cives M[3], Viggiano L[2], Lasorsa FM[2], Guarini A[1], Vegliante MC[1], Ciavarella S[1], Castellano G[7,8], Fiermonte G[2,11], Volpe G[1,9,11,12].

1- Hematology and Cell Therapy Unit, IRCCS Istituto Tumori "Giovanni Paolo II", Bari, Italy.
2- Department of Bioscience, Biotechnology and Environment, University of Bari "Aldo Moro", 70125 Bari, Italy.
3- Department of Interdisciplinary Medicine, University of Bari "Aldo Moro", Bari, Italy.
4- Max Planck Institute of Immunobiology and Epigenetics, 79108 Freiburg, Germany.
5- Experimental Pharmacology Unit, Istituto Nazionale Tumori Fondazione G. Pascale – IRCCS, Naples, Italy.
6- State Key Laboratory for Diagnosis and Treatment of Severe Zoonotic Infectious Diseases, Key Laboratory for Zoonosis Research of the Ministry of Education, Institute of Zoonosis and College of Veterinary Medicine, Jilin University, Changchun 130062, China
7- SJD Pediatric Cancer Center Barcelona, Institut de Recerca Sant Joan de Déu (IRSJD), Esplugues de Llobregat, Barcelona, Spain
8- Hematology Section, Fondazione IRCCS Cà Granda, Ospedale Maggiore Policlinico, Milan, Italy.
9- Department of Pharmacy – Pharmaceutical Sciences, University of Bari "Aldo Moro", Bari, Italy
10- Joint first authors
11- Joint Senior authors
12- Corresponding author: Prof. Giacomo Volpe, email: g.volpe@oncologico.bari.it

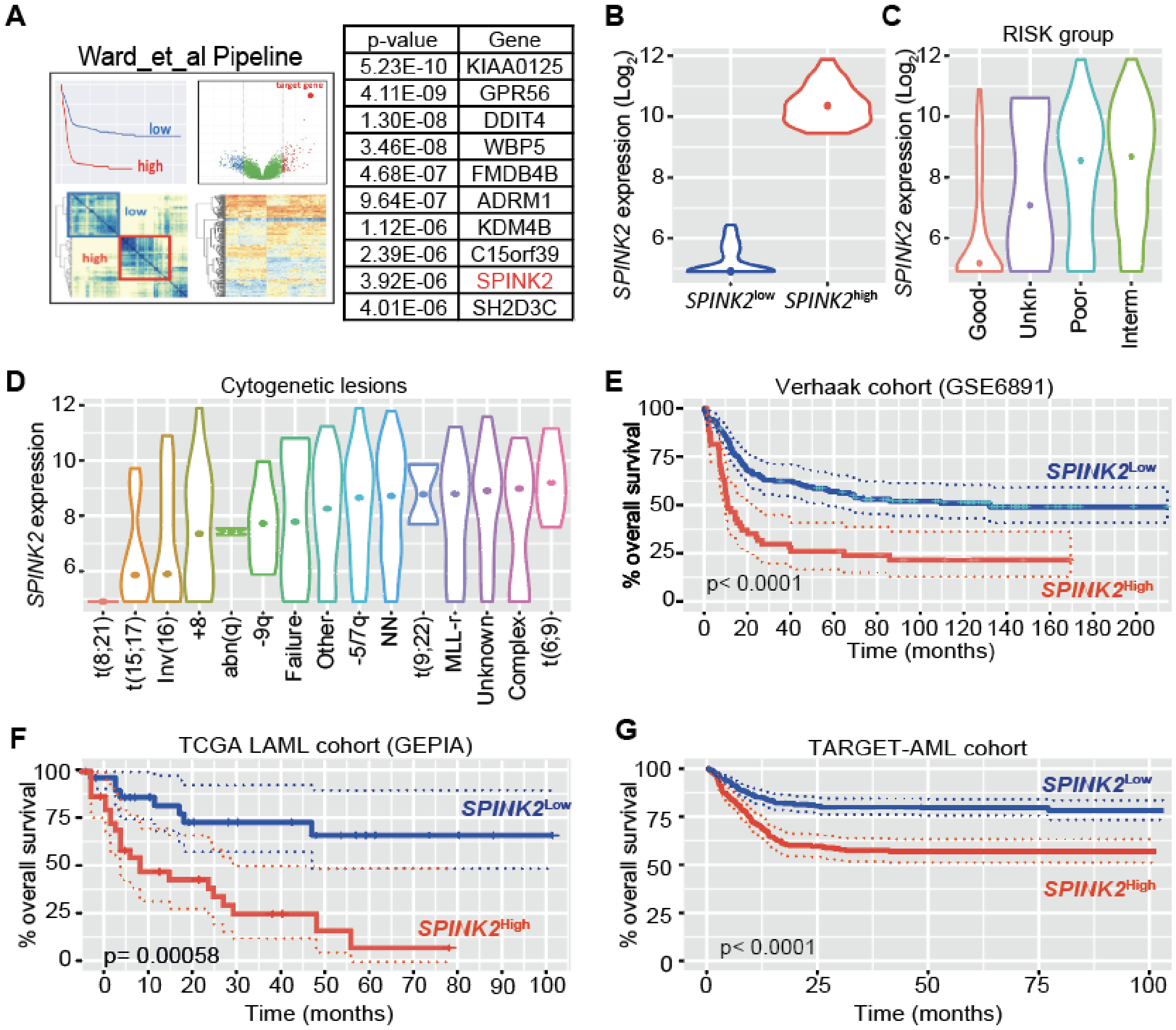


| p-value | Gene |
|---|---|
| 5.23E-10 | KIAA0125 |
| 4.11E-09 | GPR56 |
| 1.30E-08 | DDIT4 |
| 3.46E-08 | WBP5 |
| 4.68E-07 | FMDB4B |
| 9.64E-07 | ADRM1 |
| 1.12E-06 | KDM4B |
| 2.39E-06 | C15orf39 |
| 3.92E-06 | SPINK2 |
| 4.01E-06 | SH2D3C |

**Figure S1. High *SPINK2* expression correlates with inferior survival in AML patients.** (A) Schematic representation of the Ward pipeline and list of most significant genes correlated with inferior survival. (B) Violin plot showing the *SPINK2* expression levels in patients dichotomized into low and high expressers. (C) Violin plot showing the expression levels of *SPINK2* in patients belonging to different Risk categories. (D) Violin plot showing the expression levels of *SPINK2* in different karyotypic classes. (E) Kaplan-Meyer showing the overall survival of *SPINK2* low and high expressers from the Verhaak, (F) TCGA LAML and (G) TARGET-AML cohorts.

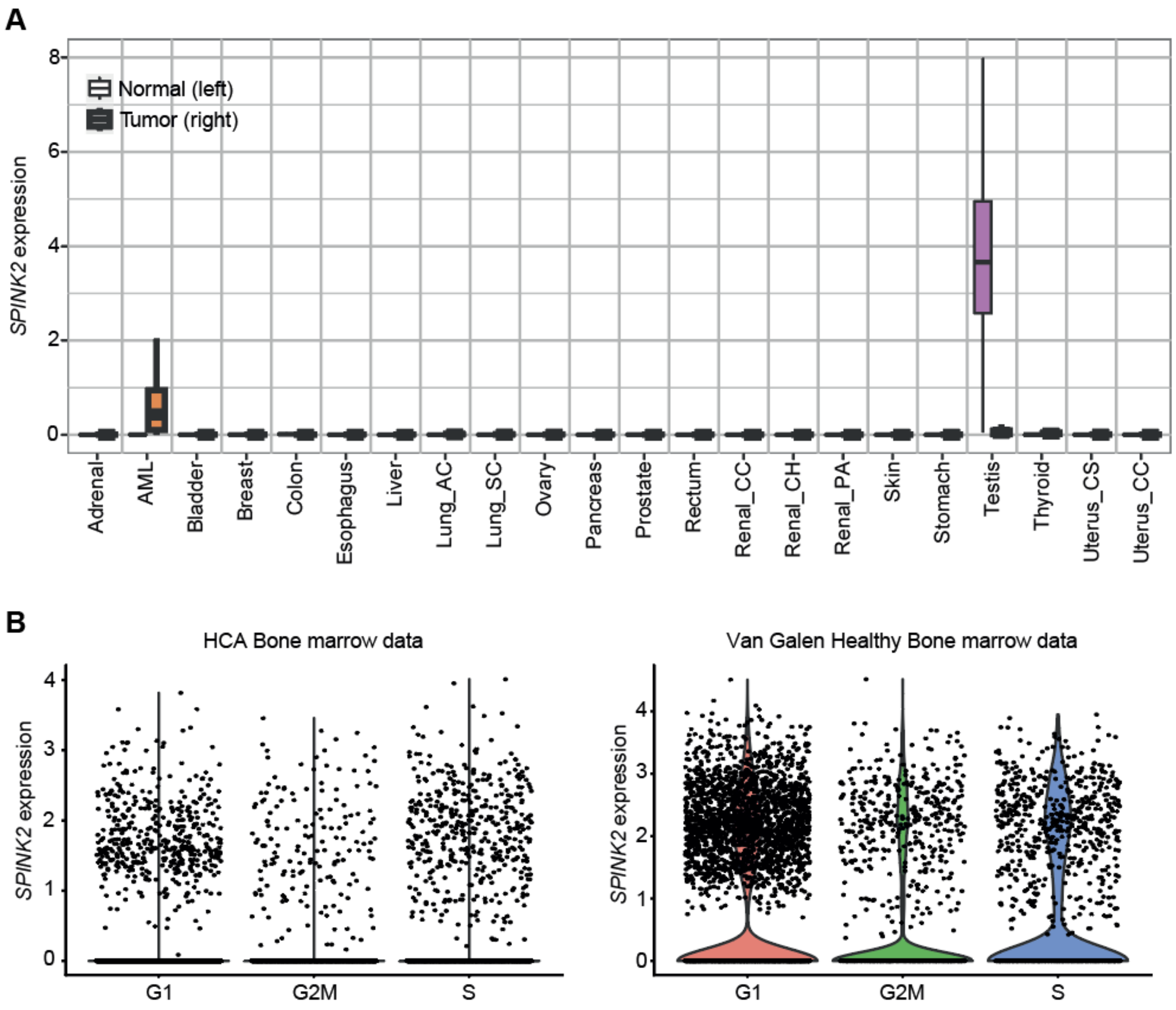


**Figure S2. *SPINK2* expression levels in different tumour types.**

(A) Boxplot showing the expression levels of *SPINK2* in health and tumour samples for different disease types. This plot was generated by the TMNplot website. (B) Violin plot showing the association of *SPINK2* expression with the different cell cycle phases in the scRNA-seq data sets of healthy human bone marrow from the Human Cell Atlas (HCA) and the Van Galen study.
